# From Monographs to Chromatograms: The Antimicrobial Potential of *Inula helenium* L. (Elecampane) Naturalised in Ireland

**DOI:** 10.3390/molecules27041406

**Published:** 2022-02-18

**Authors:** Ciara-Ruth Kenny, Anna Stojakowska, Ambrose Furey, Brigid Lucey

**Affiliations:** 1 Centre for Research in Advanced Therapeutic Engineering and BioExplore, Department of Biological Sciences, Munster Technological University, Rossa Avenue, Bishopstown, T12 P928 Cork, Ireland; ciara-ruth.kenny@mycit.ie (C.-R.K.); ambrose.furey@mtu.ie (A.F.); 2 Maj Institute of Pharmacology, Polish Academy of Sciences, 31-343 Kraków, Poland; stoja@if-pan.krakow.pl; 3 Mass Spectrometry Group, Department of Physical Sciences, Munster Technological University, Rossa Avenue, Bishopstown, T12 P928 Cork, Ireland

**Keywords:** *Inula helenium*, elecampane, antimicrobial activity, ethnobotany, compound isolation, sesquiterpene lactones, staphylococcal infection

## Abstract

With antimicrobial resistance rising globally, the exploration of alternative sources of candidate molecules is critical to safeguard effective chemotherapeutics worldwide. Plant natural products are accessible, structurally diverse compounds with antimicrobial potential. The pharmacological applications of plants in medicine can be guided by the attestation of traditional use, as demonstrated in this study. In Irish ethnomedical literature, *Inula helenium* L. (elecampane) is often indicated for respiratory and dermal ailments. This is the first assessment of antimicrobial sesquiterpene lactones from the roots of elecampane, naturalised in Ireland. Traditional hydro-ethanolic extracts were prepared from multi-origin elecampane roots. A novel clean-up strategy facilitated the bioactivity-guided fractionation of a subset of anti-staphylococcal fractions (the compositions of which were investigated using HPLC-DAD, supported by ^1^H NMR). The natural products attributing to the antimicrobial activity, observed in vitro, were identified as alantolactone **(1)**, isoalantolactone **(2)**, igalan **(3)**, and an unseparated mixture of dugesialactone **(4)** and alloalantolactone **(5)**, as major compounds. The findings suggest that the geographical origin of the plant does not influence the anti-bacterial potency nor the chemical composition of traditional elecampane root. Considering the prevalence of staphylococci-associated infections and associated broad spectrum resistance in Irish hospitals, currently, further research is warranted into the usage of the identified compounds as potential candidates in the control of staphylococcal carriage and infection.

## 1. Introduction

### 1.1. Ethnobotany in Drug Discovery

Challenges in antimicrobial chemotherapy are widespread. Most chemotherapeutic agents in clinical development today are modifications of known structures and, thus, cannot alleviate existing issues with cross- and pan-resistance among pathogens. The prevalence of antimicrobial resistance (AMR), combined with a lack of innovative leads and novel structural classes introduced to the antibacterial armamentarium in recent decades, is at the forefront of this impending crisis [1]. With ~80,000 plant species in the Amazon alone [2], the potential structural diversity in the terrestrial Plant Kingdom is enormous, albeit underutilised in medicine.

Target-directed drug discovery models have outstanding merits; however, when it comes to microbial infection, focusing on one molecule and one target could be an ineffective method over time, since microorganisms evolve at a greater rate than we can create new drugs with new targets. This is evidenced by the fact that only six new antibiotics have been approved for therapeutic use in the last 30 years and resistance has already been observed to these [3], combined with a weak pipeline for new antimicrobial agents [4]. Multi-compound (combinatorial treatment) or potentiator/adjuvant therapies [5,6] are alternatives that warrant investment.

Using an ethnobotanical approach, the pharmacological applications of plants in medicine can be guided by attesting traditional/indigenous use or by applying this knowledge to uncover natural products with new biological applications [7,8]. This approach serves as an accessible starting point and increases the probability of discovering medicinally useful compound(s), which aligns with global strategic objectives to tackle AMR [9]. Plants are a prosperous source of therapeutic chemical entities and may offer significant potential in the universal quest for effective infectious control.

### 1.2. Traditional Use to Modern Research

*Inula helenium* L. (Asteraceae family) is a distinctive perennial herb, naturalised in Ireland. The plant is a reservoir of diverse compounds, with a long history of ethnomedicinal use, with records from Minoan, Mycenaean, Egyptian, Assyrian and Serbian (Chilander Medical Codex) pharmacotherapy manuscripts circa 2700–1100 B.C. [8]. The root extract features in many traditional medical systems, including Tibetan, Ayurvedic and Traditional Chinese Medicine, where it is referred to as ‘*Radix Inulae*’ (Tu-Mu-Xiang, Zang-Mu-Xiang) [8]. In European pharmacopoeias it is referenced as ‘*Aunée’* (France), ‘*Radix Helenii’* (Netherlands), ‘*Rhizoma Helenii’* (Germany) and ‘*Helenii Rhizoma’* (U.K.) [8].

Cameron [10] postulated that *I. helenium* likely originated in Gaelic and Irish tradition, from the historic officinal name ‘*Inula campana*’, or ‘*Helénula*’ (‘Little Helen’). The plant has been referred to in traditional Irish texts as ‘*áillean*’, derived from ‘*áille*’ meaning beautiful or lovely, and ‘*Ellea*’ derived from the Gaelic ‘*Eilidh*’ meaning Helen [10]. This association is thought to be rooted from Greek Mythology, as Helen of Troy favoured the flowers of *I. helenium* for their aesthetic splendour [10]; as legend recites, the blossom of elecampane from her fallen tears upon abduction from her homeland [11]. The plant features in Celtic folklore, as ‘*Elf-Dock’* and ‘*Elf-Wort*’, and other common names include ‘*Horse-heal*’ and ‘*Scab-wort*’; the latter relating to its use as a topical agent [11].

In ancient Irish literature, the medicinal use of *I. helenium* is often described for respiratory ailments [12]. In a translated version of Tadgh Ó Cuinn’s medieval Irish *Materia Medica* (circa 1415 A.D.), references to the use of *I. helenium* to treat respiratory organs, coughs and consumption [from tuberculosis], are documented. Respiratory preparations specified boiling the powdered herb with dilute barley water, liquorice, cinnamon and sugar, whilst digestive ailments (e.g., “*ileus, colic and stranguria*”) were treated with a plaster applied to the naval, comprised of the herb boiled in wine and oil [12]. Celtic ethnobotany is somewhat historically neglected, however, and as Moloney [13] recites: “[*we have*] *relegated to oblivion many a(n) herb*”. The recent establishment of digital archives (e.g., Dúchas.ie, CELT.ie) will function to preserve and facilitate further research of Irish traditional medicinal knowledge [14,15].

The British Herbal Pharmacopoeia (BHP) lists the therapeutic actions of the herb, as follows: antitussive, antiseptic expectorant, diaphoretic and bactericidal [16]. Suggested indications include respiratory mucosal catarrh (tracheal, bronchial), cough and phthisis associated with pulmonary tuberculosis, bronchitis and whooping cough in infants [16]. The root is traditionally administered as a decoction, prepared by boiling the comminuted herbal material and water, which is then strained before administration [17]. Dosage recommendations in the BHP range from 1.5–4 g decoction from the dried root/rhizome, or 1.5–4 mL liquid extract (i.e., 1:1 herbal tincture in 25% alcohol) thrice daily [16]. The plant can be combined with extracts of *Marrubium*, *Tussilago, Asclepias* or *Millefolium* [16], however, guidelines for such preparations are not detailed in the monograph.

In the 1980s, the German Commission E published an official monograph disproving the therapeutic use of elecampane root, based on insufficient evidence to support the efficacy of the herb and preparations thereof, and the risk of associated adverse effects [18]. It was later considered an “unapproved” medicinal herb, and consequently excluded in consecutive global monographs and compendial (Pharmacopeial) texts from thereon, including the following: the ESCOP monographs, WHO Selected Medicinal Herb monographs (Vols. 1–3), the British Herbal Compendium (Vols. 1 and 2), and the European Medicines Agency (EMA) official herbal monographs and list entries. This outcome was a likely consequence of individual case reports, documenting adverse inflammatory effects thought to be associated with *I. helenium* [19,20,21,22] and related species (*I. conyza* [23,24], *I. viscosa* [25,26] and *I. graveolens* Desf.) [27]. The findings of these earlier studies accept that the presence of causative allergens was likely family/genus related. Paulsen [28] acknowledges that while elecampane is suspected to be an inducer of Asteraceae allergic dermatitis, there is no epidemiological data available to support this. Sesquiterpene lactones (SLs) are an important group of bioactive metabolites present in elecampane root [8,29], as shown in Table 1. Paulsen [30], in agreement with Amorim [31], later cautioned towards the systemic allergic dermatitis associated with SL-containing plants in general, while emphasising the need to determine the pathogenesis of haptens, associated with the plant species, more specifically. Clinical data for the herb is limited, with one randomised, double-blind, placebo-controlled clinical trial documented for a cough syrup (KalaboTUSS^®^), which proved to be safe and efficacious [32]. Regarding isolated compounds, there are conflicting reports of sensitization [33,34,35] and opposing anti-allergenic properties [36,37]. More recent evidence, however, infers support for further research into the therapeutic efficacy and safety of elecampane-derived compounds.

Research interest has increased in recent years, specifically relating to the potential of the crude extract and isolated compounds (viz. alantolactone, isoalantolactone, 5α-epoxyalantolactone, (iso) costunolide and igalan) in cancer therapeutics. Antineoplastic [38] and anti-cancer activity was demonstrated in multiple cell lines, including brain [39], pancreatic [40] and breast [41] cancers. Other studies reported reduced inflammatory responses in in vivo sepsis models [42,43], rheumatoid arthritis [44] and suppressed neutrophil-mediated inflammation in acute bronchitis by down-regulating the β2-integrin [45], following exposure to the crude extract. Alantolactone has been reported to exert anti-tumour activity in a number of cancer models, including Jurkat T-lymphocyte cells [46], β-cell acute lymphoblastic leukaemia [47], chronic myelogenous leukaemia [48], gastric [49], colon [50,51], liver [52,53], lung [54,55,56], pancreatic [57] and breast cancers [58,59]. Moreover, the compound inhibited lipopolysaccharide (LPS)-induced nitric oxide synthesis in murine macrophages (RAW 264.7), the activity of which is attributed to the presence of the α-methylene-γ-lactone moiety—a key structural motif and putative pharmacophore of many identified SLs [60].

Isoalantolactone exerts diverse anticancer activity in vitro [61], including squamous cell carcinoma (head, neck) [62], oesophageal cancer [63], chronic myelogenous leukaemia [64] and breast cancer cell lines [65]. It inhibits LPS- and Phorbol-12-myristate-13-acetate (PMA)-induced inflammatory responses [66,67]. In pulmonary models, it exerts anti-inflammatory effects in acute lung injury (ALI) [68] and inhibits α-toxin, an important virulence factor secreted by *S. aureus*, which potentiates pneumonia pathogenesis [69]. Isoalantolactone, alantolactone and alloalantolactone have comparable anti-tumour effects in pancreatic cell lines [70]. Alantolactone [71], isoalantolactone [72] and 5α-epoxyalantolactone were reported as potential chemopreventative agents [73], while 5α-epoxyalantolactone exhibits antiproliferative effects in acute myelogenous leukaemia progenitor cells [74]. Igalan demonstrated protective [75] and anti-inflammatory activity in vivo [76]. Lastly, costunolide and isocostunolide, neither of which were identified in this study, also show promise for the anti-proliferative, anti-metastatic and neuroprotective effects in vivo [77,78,79].

### 1.3. Antimicrobial Potential of Elecampane SLs

Olechnowicz-Stepien and Skurska [80] first reported the antimicrobial activity of the root in vitro, while some of the earliest compounds isolated included alantopicrin [81] and dammaradienyl acetate [82]. A cascade of research followed in the late 1990s, investigating the antimicrobial activity of different extracts of elecampane root against various pathogens [43,83,84,85,86,87,88,89,90,91,92]. Methicillin- and vancomycin-resistant *Staphylococcus aureus* (MRSA/VRSA) are among the listed pathogens of “medium-to-high priority”, published by the WHO [1], and at a national level, Ireland has some of the highest occurrences of *Staphylococci*- and *Enterococci*-associated systemic bloodstream infections in all the European member states [93]. *S. aureus* was, therefore, selected as a suitable target organism in this present study, because of its priority status and clinical relevancy in Irish hospitals. Furthermore, it is in-line with ethnobotanical use and the microorganism has a known susceptibility to crude extracts of elecampane, as previously demonstrated in our laboratory [86], and among other research groups, as summarised previously [3].

The aim of this study is to complete the first assessment of antimicrobial SLs in a traditional (hydro)ethanolic root extract of elecampane, naturalised to the Irish climate, with comparison to dried root sourced internationally. Documented here is the application of a novel one-step clean-up strategy to facilitate the bioactivity-guided fractionation of antimicrobial compounds, attributing to the anti-staphylococcal activity observed in vitro. A validated HPLC-DAD method was used to investigate the presence of alantolactone **(1)**, isoalantolactone **(2)**, igalan **(3)** and an unseparated mixture of dugesialactone **(4)** and alloalantolactone **(5)**—which could serve as potential antibiotic lead compounds. The findings suggest no observed difference in antimicrobial activity from extracts of different origin, suggesting the antimicrobial activity of elecampane is likely species-specific and is independent of the cultivation environment.

**Table 1:** Overview of known isolated sesquiterpene lactones (SLs) from *I. helenium* root extracts to date.

Group	No.	Identified Compound(s)	Reference(s)
Eud-	1	Alantolactone	[94,95,96,97,98]
	2	Isoalantolactone	[99,100,101]
	3	Dihydroalantolactone	[102,103,104,105]
	4	Dihydroisoalantolactone	[102,103,104,105]
	5	Tetrahydroalantolactone	[106]
	6	Alloalantolactone (= 1-Deoxyivangustin, = (+)-Diplophyllin)	[107]
	7	Bialantolactone	[108]
	8	Trinoralantolactone	[108]
	9	5α-Epoxyalantolactone	[107]
	10	4-Noralantolactone (= 4-oxo-5(6),11-eudesmadiene-8,12-olide)	[109]
	11	4-Norisoalantolactone (= 4-oxo-11-eudesmene-8,12-olide)	[109]
	12	1α-Hydroxy-11,13-dihydroisoalantolactone	[110]
	13	3α-Hydroxy-11,13-dihydroalantolactone	[110]
	14	Macrophyllilactone E	[110]
	14	4α,15α-Epoxyisoalantolactone	[108]
	15	4,5-seco-Eudesm-11(13)-en-4,5-dioxo-8β,12-olide (=Umbellifolide)	[108]
	16	11α-Hydroxyeudesm-5-en-8β,12-olide	[108]
	17	3α-Hydroxyeudesma-4,11-dien-8β,12-olide	[108]
	18	Telekin	[108]
	19	3-Oxo-eudesma-4(5),11-dien-8,12-olide (= 3-Oxoalloalantolactone)	[111]
	20	11α,13-Dihydro-α-cyclocostunolide	[112]
	21	11α,13-Dihydro-β-cyclocostunolide	[112]
	22	15-Hydroxy-11βH-eudesm-4-en-8β,12-olide	[112]
	23	3α-Hydroxy-11βH-eudesm-5-en-8β,12-olide	[112]
	24	2β,11α-Dihydroxy-eudesm-5-en-8β,12-olide	[112]
	25	Isoheleproline	[113]
	26	11β-Hydroxy-13-chloro-eudesm-5-en-8β,12-olide	[7]
	27	5-epi-telekin	[7]
	28	Racemosalactone A	[7]
	29	Macrophyllilactone F	[74]
El-	30	Igala (= 1,3,11(13)-Elematrien-8β,12-olide)	[105]
Er-	31	Dugesialactone	[114]
Gua-	32	Dehydrocostus lactone	[112]
	33	4α-Hydroxy-1β-guaia-11(13),10(14)-dien-12,8α-olide	[111]
Ger-	34	Germacrene-D-lactone (= Germacra-1(10),4(15),5(6),11(13)-tetraen-8,12-olide)	[107]
	35	4β,5α-Epoxygermacra-1(10),11(13)-dien-12,8α-olide	[105]
	36	Isocostunolide	[79]
	37	(1(10)E)-5β-Hydroxygermacra-1(10),4(15),11(13)-trien-12,8α-olide	[109]
	38	14-Hydroxy-11β,13-dihydrocostunolide/ 11β, 13-Dihydro-14-hydrocostunolide	[8,112]
	39	Costunolide	[112]
	40	5β-Hydroxygermacra-1(10),4(15),11(13)-trien-12,8β-olide	[108]
	41	4α,5α-Epoxygermacra-1(10),11(13)-dien-12,8β-olide	[108]

Eud-: Eudesmanolides; El-: Elemanolides; Er-: Eremophilanolides; Gua-: Guaianolides; Ger-: Germacranolides.

## 2. Results and Discussion

There is widespread acceptance that AMR presents alarming dangers, and the current clinical antimicrobial pipeline is insufficient to allay consequent threats to global infection prevention and control [1]. This reality has resulted in a united global ambition in the pursuit of new therapeutic modalities for infectious disease. Plants documented as anti-infectives in historical literature, such as *I. helenium*, could offer potential as mono-/poly-therapeutics and adjuvant/potentiator agents in modern medicine [5,6]. The use of plants and Ethnobotanical principles as a starting point in the drug discovery process, therefore, warrants consideration. The aim of this study was to investigate the key compounds from a traditional (hydro)ethanolic root extract of *I. helenium,* attributing to its in vitro anti-staphylococcal activity.

An initial preliminary screen confirmed that the crude extracts were active against the gram-positives, as follows: Group-A *Streptococcus pyogenes*, Group-B *Streptococcus agalactiae*, *Listeria monocytogenes*, *Escherichia faecalis* ATCC 29212 and *Escherichia coli*, as well as *Mycobacterium tuberculosis* H37Ra (ATCC 25177) (data not shown). *S. aureus* was chosen as the target organism for the bioactivity-guided fractionation in this study, based on previous results [86], and its clinical relevance in Irish hospitals currently [93].

**Figure 1:**
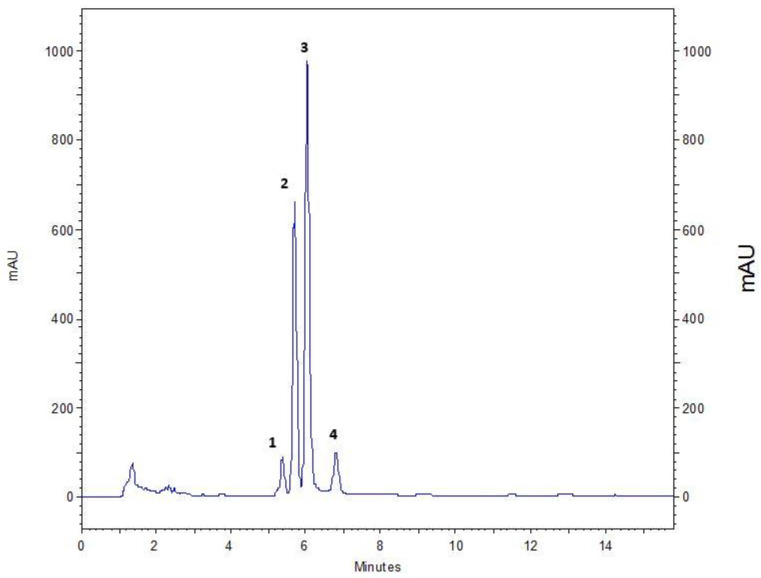
Chromatographic separation of the fraction F16 (CM50) by the method proposed by Huo et al. [114], using the Zorbax^®^ Eclipse XDB C_18_ column. Signals detected at 210 nm: Peak 1—igalan; 2—isoalantolactone; 3—alantolactone; 4—a mixture of dugesialactone and alloalantolactone.

**Figure 2:**
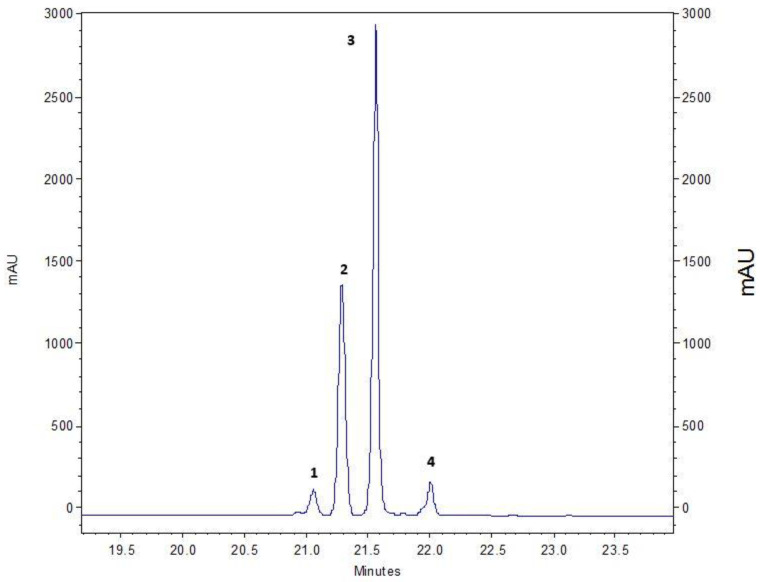
Chromatographic separation of the fraction F16 (CM50) using the method proposed by Stojakowska et al. [119], using the Kinetex^®^ 5 μm XB-C_18_ column. Signals detected at 205 nm: Peak 1—igalan; 2—isoalantolactone (confirmed with standard); 3—alantolactone (confirmed with standard); 4—mixture of compounds containing alloalantolactone (confirmed by ^1^H NMR).

An optimised extraction method, involving the traditional maceration of the comminuted root with the extractant solvent, was performed as before [86], to compare the activity between the root originating from a source plant naturalised to West Cork (Ireland) versus internationally sourced, commercially available dried root samples. The compounds attributing to the bioactivity, observed in vitro, were identified as alantolactone **(1)**, isoalantolactone **(2)**, igalan **(3)**, and an unseparated mixture of dugesialactone and alloalantolactone **(4, 5)**, as major constituents. Our findings suggest that the antimicrobial potency of the plant extract is not influenced by geographical origin or environmental conditions in this case, since all samples resulted in comparable activity (See Table 2) and major constituent profiles (See Figure 1 and Figure 2).

As a follow-on from our preliminary elecampane research [86], the bioactive composition of the crude extract was further explored in this study. This was achieved using an in vitro bioactivity-guided fractionation strategy, applied to fractions generated from a single chromatographic clean-up step, using Sephadex LH-20 as the stationary matrix. This model could serve as a starting point for small-scale process development, if considering the general extraction of antimicrobial compounds from this plant. Sephadex LH-20 was the matrix of choice based on the desire to minimize compound loss via in situ sorption effects/phenomena. Sephadex LH-20 has been used for the isolation of compounds from other *Inula* species [115,116,117,118]; however, this is the first account of its use for the initial fractionation of bioactive compounds from crude extracts of *I. helenium*. Results from the agar-well screening suggested that the composition of the active fractions contained a range of compounds that were physiochemically related and hence co-eluted within a narrow range, e.g., F16–24 (See Table 3).

**Figure 3:**
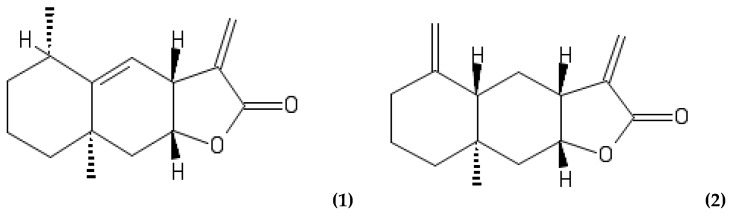

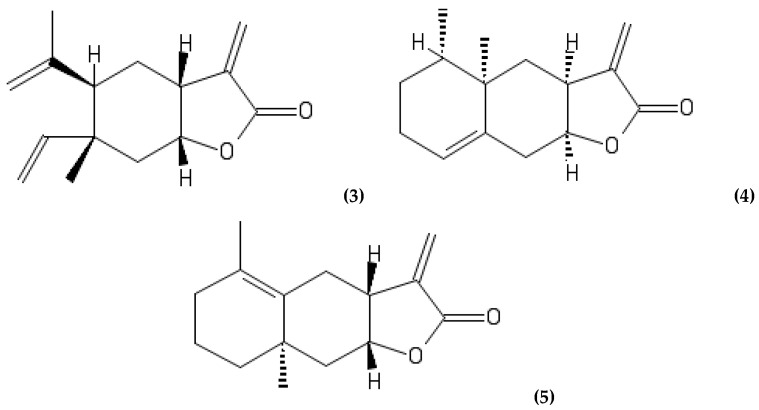
Structures of the identified compounds: (**1**) alantolactone; (**2**) isoalantolactone; (**3**) igalan; (**4**) dugesialactone and (**5**) alloalantolactone.

Further elucidation of the composition of the active fractions was performed, following methods routinely used at the Phytochemistry Department of the Maj Institute of Pharmacology, for quantification of SLs in various extracts from plants of the *Inuleae* tribe [119]. Figure 1 and Figure 2 outline the HPLC chromatograms for fraction F16 (CM50), following the Huo et al. [114] and Stojakowska et al. [119] methods, respectively. Since both methods produced comparable spectra, the Stojakowska et al. method [119] was used exclusively. All samples, regardless of their source location, similarly contained a mixture of closely related eudesmanolides (i.e., helenin) with alantolactone (1) and isoalantolactone (2) as major signals—annotated in Figure 1 and Figure 2 as chromatographic peaks 2 and 3—and confirmed with comparison to external standard samples as per [119]. Structures of the identified SLs are shown in Figure 3.

Quantitatively, the mixture of the eudesmanolides constituted up to 50% of the sample weight (See Table 3). To confirm the identification of the partially overlapping minor lactones (Peak 4, Figure 2), ^1^H NMR was performed, as per Huo et al. [114], as shown in the Supplementary Data (Figure S4). Peak assignments were consistent with those available in the literature [109,114], however, ^1^H NMR analyses of subfractions were influenced by the presence of lipids/fatty acids (See Table 3). The content of the mixture under Peak 4 was assessed semi-quantitatively, with an assumption that the signal is generated by eudesmanolides (Compounds 4 and 5).

SLs, depending on the structure of their carbon skeleton, can be classified into several groups, including germacranolides, eudesmanolides, guaianolides and pseudoguanianolides. A comprehensive list of known SLs, identified in *I. helenium* root, can be found in Table 1. Several reviews discuss the extensive pharmacological potential of metabolites from the *Inula* genus [3,8,29,120,121].

There are, however, limitations to this study. Natural product extracts are complex, comprised of multiple compounds of unknown molecular weight and variable characteristics (e.g., polarity, solubility, viscosity, stability, toxins, fluorophores, pigments) that can cause bioassay interference in manual and automated screening platforms [122,123]. Furthermore, the unavailability of standardised antimicrobial breakpoints to guide AST of natural products, such as plant compounds, is a recognised limitation in this area of research [3]. The crude extract of *I. helenium* ranges from green (aqueous-ethanolic extract) to dark brown (ethanolic extract). Both the plant extract pigmentation and the extractant solvent are central to the experimental issues our lab observed when performing preliminary dilution-based antimicrobial methods, including microdilutions (i.e., MIC determination) and biofilm assays (data not reported). Pigmentation of extracts/fractions can interfere with spectral quantification of microbial turbidity and biofilm staining, irrespective of the use of colorimetric indicators. Some authors report the pre-treatment of coloured plant extracts, prior to testing, such as the multiple sequential centrifugation of the crude filtrate [124], or the addition of decolourisation steps (e.g., activated carbon) for the removal of non-polar pigments [125]. Any pre-treatment in the sample preparation phase would need to be considered, of course, when interpreting the results, to avoid adversely affecting intra- and inter-laboratory reproducibility [126].

Solvents are employed for the initial extraction, chromatographic separation and re-solubilisation of dried plant residues. In this study, EtOH was chosen as the extractant solvent, based on traditional usage [86], and similar studies evaluating EtOH extracts of *I. helenium* [88,109,110,127,128]. In dilution-based methods, solvents can interfere with activity—either potentiating or falsely depicting antimicrobial activity. Increased dilution ranges, using assay media as the diluent, are, therefore, necessary, which can sometimes lead to an underrepresentation of activity, since water-based diluents can be inefficient vehicles for compound solubility [129]. Solubilisation of the test articles, across a wide range of polarities, in an inert or biologically inactive solvent that is miscible with assay media is preferred [130]. Cyrene^TM^ (dihydrolevoglucosenone), an aprotic dipolar solvent, has recently been proposed as a green bio-based alternative to DMSO, offering comparable solubility properties, low toxicity and no evidence of antioxidant or radical scavenging properties, unlike DMSO [129]. Validation of its use in phytochemical research would be valuable to this field.

A negative control containing the solvent, or carrier, used to extract or dissolve the test article(s), was included in order to confirm inactivity and non-toxicity against the target microorganism. We performed a solvent tolerance test and confirmed that our target organism tolerated EtOH, up to 20% per well in the microdilution method (data not shown). With the diffusion-based assay, the EtOH content of the test articles (e.g., crude extracts, fractions or standards) did not adversely affect the assay, as the plates were left under laminar flow to evaporate the EtOH prior to incubation. Similarly, the pigment was retained within the well and did not diffuse throughout the agar. The evaluation of dilution and diffusion methods, used to determine the antimicrobial activity of plant extracts, has been reviewed [3,130]. The EUCAST guidelines, while directed for single-constituent or conventional antibiotics, encompass the core criteria to perform AST, including inoculum preparation, media preparation and dilution schemes and were, therefore, used in this current study, in the absence of any standardised methods for antimicrobial assessment of plant extracts. Outside conventional AST, methods exploring other aspects of bacterial weaponry constitute a relatively untapped market for novel targets to complement anti-infective strategies, such as methods targeting virulence factors (e.g., adhesins, toxins, effectors, ion chelators, destructive enzymes, secretory and signalling molecules), structural assembly, and biofilms compositions [131].

Our results demonstrate that in vitro anti-staphylococcal efficacy of a traditional extract of *I. helenium* root is in line with traditional use [12,16]. Its use in dermal (topical) applications is minimally explored in recent scientific literature, which is a likely consequence of earlier anecdotal case reports of species and family-related sensitivity. Given the prevalence of *Staphylococci*-associated infections in Irish hospitals, further research into the usage of the identified compounds (alone and in combination) is warranted as an alternative treatment in the prevention and control of *Staphylococcal* carriage and infection.

## 3. Materials and Methods

### 3.1. Crude Extract Preparation

#### 3.1.1. Sources of Plant Material

**Figure 4:**
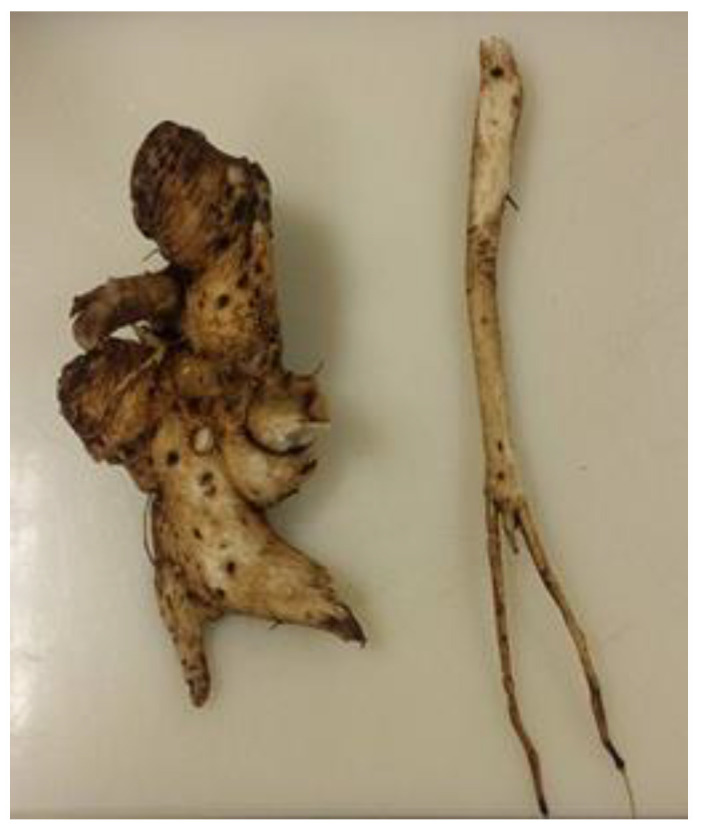
Harvested roots of *I. helenium* L. (Elecampane).

Cultivated roots (CT) were collected from Bandon Medicinal Herbs Ltd. (West Cork, Ireland) (See Figure 4), and authenticated accordingly [86]. The plant material was also identified against a voucher specimen deposited in the New York Botanical Gardens (NYBG) Steere Herbarium (Barcode: 2924501), and botanical key [132]. Commercial dried root (CM) was purchased from a registered supplier, Herbs in a Bottle© Ltd. (U.K.). Product information as per label: Code #6202b; Batch #148845; Specification: Cut; Date of Manufacture: 18/06/2015; Issue #160575; Origin: China; Identification: Conforms.

#### 3.1.2. Traditional Maceration

Roots and rhizomes were collected in September 2015 (Bandon Medicinal Herbs Ltd.). The harvested roots were washed with ultrapure Milli-Q water (18.2 MΩ·cm) and left to dry naturally at room temperature. The dried roots were then powdered and stored in a sterile air-tight container protected from light.

**Figure 5:**
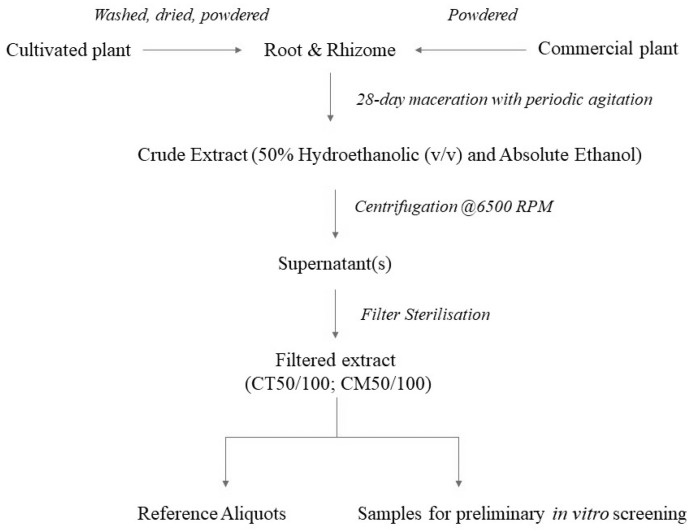
Schematic overview of the extraction process.

**Figure 6:**
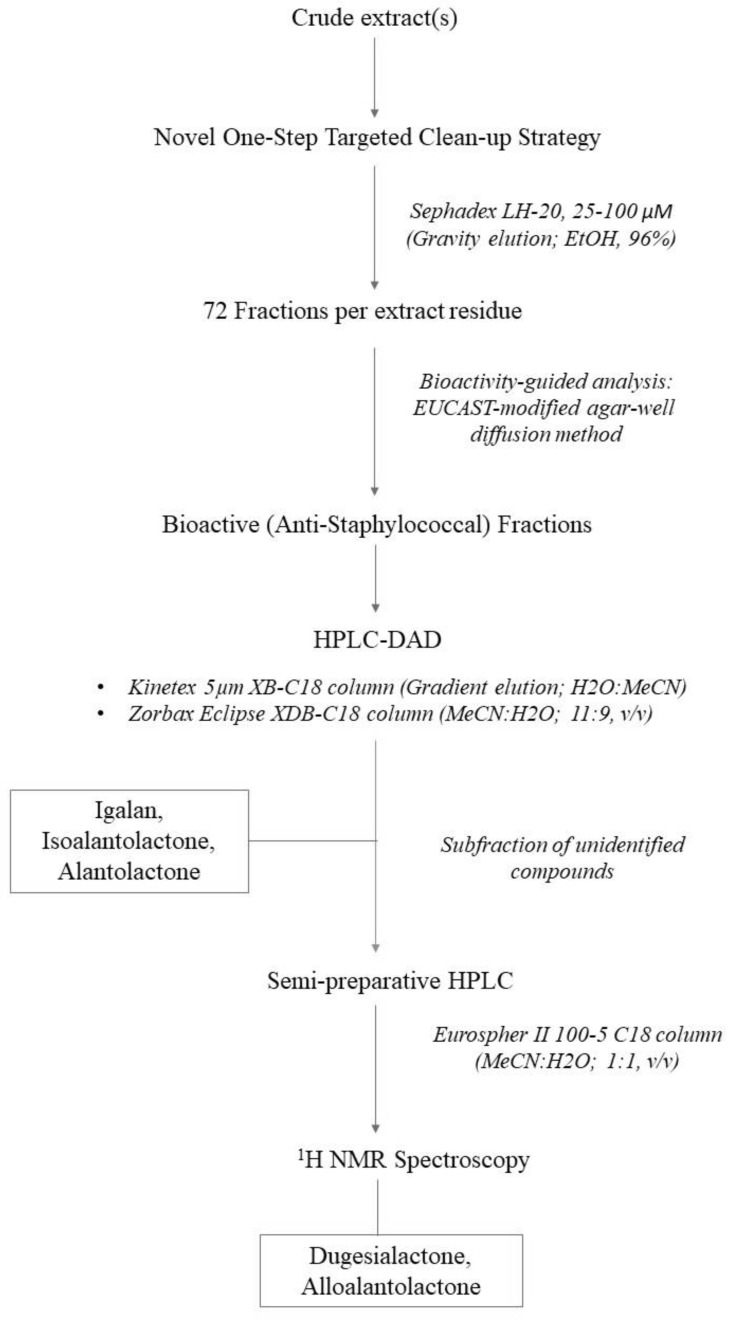
Schematic overview of the phytochemical analysis methodology.

To compare cultivated versus commercially sourced plant material, a total of four plant extracts were prepared at a concentration of 100 mg mL^−1^ in 50% aqueous ethanol (*v*/*v*) and absolute ethanol (See Table 4), as per O’Shea et al. [86]. The extracts will be referred to as CT50 or CT100 (cultivated), and CM50 or CM100 (commercial), from herein. See Figure 5 and Figure 6 for schematic overviews of the extraction and analysis process.

The comminuted herbal material was introduced to the vessel containing the extraction solvent as per Table 4. Extracts were periodically mixed by gentle inversion. Maceration took place for a total of 28 days at room temperature protected from light. The crude macerate(s) were centrifuged in a Thermo Scientific IEC-CL30R for 15–20 min at 6500 RPM. The decanted supernatant(s) were subsequently vacuum filtered using a Büchner funnel lined with Whatman filter paper No. 1 discs for clarification (11 µm particle retention) followed by filter sterilisation using cellulose nitrate loaded syringes (0.45 µm). Retained samples of the crude extracts were stored at −20 °C in a repository at Muster Technological University, while the test articles were stored at 2–8 °C for use as an experimental control.

### 3.2. Bioactivity-Guided Fractionation of Antimicrobial Compounds

#### 3.2.1. Gravity-Eluted Size-Exclusion Chromatography

The solvent removal process was performed using a BÜCHI Rotavapor R-205 (BTX-RE2) with an attached BÜCHI vacuum controller V-800. The instrument parameters were set (40 °C; 175 mbar) and total evaporation time was approximately 90 min per 100 mL extract. Total yields are recorded in Table 4. Residues were stored at −20 °C until required for further analysis.

The CT50 extract was retrieved from −20 °C storage and left to thaw at room temperature. Methanol (MeOH) was added to resolubilise the viscous residue under sonication. The resuspended extract was transferred to an amber vial (2 mL) and the solvent was removed under a gentle flow of nitrogen. Ethanol (EtOH; extractant solvent) was used to resolubilise the extract as it is less potently toxic to cells in vitro. To maintain consistency, EtOH was further utilised as the mobile phase for subsequent chromatographic separation.

Sephadex LH-20 (25–100 µm) was prepared in MeOH and left in solution for 24 h at room temperature. All glassware was rinsed in MeOH and air-dried before use. A glass column (height 90 cm; diameter 3.2 cm) was carefully packed with the Sephadex solution and secured with parafilm to facilitate deposition. The Sephadex was prevented from drying out in the column once packed by ensuring that the apex remained submerged in EtOH. The soluble crude extract (2 mL, 100 mg) was applied to the column as a thin solvent band using a glass pipette, and the band was allowed penetrate the top layer. Once completed, a reservoir of EtOH (300 mL) was introduced as the mobile phase. The column was eluted with 96% EtOH under gravitational flow, and fractions were manually collected in 10 mL aliquots. A total of seventy-two fractions were collected per extract over a period of 12 h.

These steps were repeated for the remaining three following extracts: CT100, CM100 and CM50. All fractions were immediately screened for their antimicrobial activity against *Staphylococcus aureus* using a modified in vitro agar-well method, detailed below.

#### 3.2.2. Bacterial Strains and Media Preparation

- Clinical diagnostic reference strain *S. aureus* NCTC 6571 (cross-referenced in the American Type Culture Collection (ATCC) as ATCC 9144 [133]).
- *S. aureus* clinical isolates from Cork University Hospital (CUH), Co. Cork, Ireland.
- Culture media prepared as per manufacturer guidelines: Mueller Hinton (MH) broth (Lab M, Lancashire, U.K.; Lot: 141370/357) and agar (Lab M, Lancashire, U.K.; Lot: 144209/172). Cation-adjusted Mueller Hinton II (MHII) broth (Sigma-Aldrich, Darmstadt, Germany; Lot: BCBT9094) and agar (Sigma-Aldrich, Darmstadt, Germany; Lot: BCBV4646).
- Sodium chloride (PanReac AppliChem, Barcelona, Spain; Lot: 0000893728).
- Glycerol solution, 84–88% (Sigma-Aldrich, Darmstadt, Germany; Lot: SZBC010BV).
- Alantolactone standard (Sigma, Darmstadt, Germany; Lot No. 125M4751V).
- Isoalantolactone standard (CliniSciences; HY-N0780/CS-3635; Batch No. 20994).

#### 3.2.3. Preparation of *S. aureus* Stocks

*S. aureus* was used as the target organism to guide the fractionation process. Stocks were maintained in glycerol at −80 °C. Briefly, overnight cultures were centrifuged at 4000 RPM for 15–20 min. The supernatant was discarded, and the pellet re-suspended in MH broth. Aliquots were combined with sterile glycerol (1:2, *v*/*v*) under laminar flow. Working stocks were stored at −20 °C and reference stocks at −80 °C.

#### 3.2.4. In Vitro Agar-Well Screening (Modified EUCAST Disk-Diffusion Method)

Antimicrobial screening was performed following European Committee on Antimicrobial Susceptibility Testing (EUCAST) Guidelines [126,134], modified accordingly. Briefly, overnight cultures of *S. aureus* NCTC 6571 were adjusted to 0.5 McFarland standard in 0.85% sterile saline solution. The surfaces of MH agar plates were inoculated with the adjusted bacterial suspension using sterile cotton swabs. To prepare the wells, 8 mm holes were aseptically punched into the agar surface after inoculation. Seventy-five µL of each fraction were transferred to their corresponding well. Alantolactone (0.2–3.2 µg mL^−1^) and isoalantolactone (1 mg mL^−1^) were tested in tandem. Plates were left under laminar airflow for up to 60 min to facilitate solvent evaporation prior to incubation at 37 °C. Mupirocin and crude elecampane extracts were used as positive controls, and sterile water as a negative control. Plates were prepared in triplicate and incubated for 24 h at 37 °C. Antimicrobial activity was determined by measuring zones of inhibition (mm; $\bar{x}$ (mean) ± SD (standard deviation)) using calibrated Vernier callipers (Mitutoyo).

#### 3.2.5. Solvent Tolerance Test

To assess the possibility of EtOH potentiating antimicrobial activity in the microdilution method, a separate test was used to confirm inactivity using a solvent tolerance assay as per [135] with modifications. Eleven strains of clinical *S. aureus* isolates, including methicillin-resistant *S. aureus* (MRSA), were used as the target organism. The EtOH concentration range increased in 1% increments (1–30% EtOH). A 10% addition of AlamarBlue^TM^ was added to the wells before measuring absorbance.

### 3.3. Structural Investigation of Bioactive Fractions

#### 3.3.1. Sample Preparation

Each bioactive fraction was reduced to dryness using a Zymark^TM^ TurboVap^®^ LV Concentration Evaporator system. Nitrogen flow rate was set to 20 psi for 20 min and increased incrementally to 50 psi as volume reduced. Each dried residue in the test tube(s) was resolubilised in pure EtOH, sonicated and transferred to amber HPLC vial(s) (1 mL in total). The samples were reduced under a gentle flow of nitrogen again and stored at −20 °C until required for further analysis.

#### 3.3.2. Standards and Reagents

External standards asperilin (purity > 95%) and isoalantolactone (purity > 90%) were isolated from flowers of *Telekia speciosa* (Schreb.) Baumg., as it was described earlier [119]. A mixture of alantolactone and isoalantolactone of lower purity, isolated from *I. helenium* roots, was also used for identification purposes. Water was purified by a Milli-Q system (Millipore Corp, Bedford, Massachusetts, USA). MeOH and acetonitrile (MeCN) of gradient grade for liquid chromatography were purchased from Merck (Darmstadt, Germany).

#### 3.3.3. HPLC-DAD Analysis

Chromatographic separations were performed using Agilent 1200 Series HPLC system (Agilent Technologies, Palo Alto, CA, USA) equipped with a Rheodyne manual sample injector, quaternary pump, degasser, column oven and a diode array detector (DAD). Analytical separations were carried out using a Kinetex^®^ 5 μm XB-C_18_ column (260 × 4.6 mm, 100 A pore size) from Phenomenex (Torrance, CA, USA), at 40 °C, with a gradient mode elution, as it was described elsewhere [119]. The mobile phase consisted of H_2_O (A) and MeCN (B). Linear gradient from 12% B to 15% B in 5 min, 25% B in 5 min, 60% B in 5 min, 98% B in another 10 min was applied (stop time: 35 min; post time: 12 min). Flow rate was established at 1 mL min^−1^. Alternatively, a Zorbax^®^ Eclipse XDB-C_18_ column (150 × 4.6 mm, 5 μm) (Agilent, Santa Clara, CA, USA) with a mobile phase consisting of MeCN and H_2_O (11:9, *v*/*v*) was used, as it was proposed by Huo et al. [114]. As similar chromatographic resolutions of the analysed compounds were achieved by both methods, the gradient elution system was used exclusively. This method is routinely used at the Phytochemistry Department of the Maj Institute of Pharmacology at the Polish Academy of Sciences (PAS), for quantification of SLs in various extracts from plants of the *Inuleae* tribe.

Accurately weighted aliquots of the active samples were transferred into 1.5 mL Eppendorf tubes and dissolved in 1 mL of 70% MeOH and MeCN mixture (1:1, *v*/*v*). The solution was centrifuged (11,340 g, 5 min) prior to HPLC analysis, and injected (5 μL) into the column. The detection wavelength was set at 205 nm. Quantification of compounds 1–3 was done by an external standard method (ESM), as it was described earlier [119]. The content of the mixture under Peak 4 (Figure 2) was assessed semi-quantitatively, with an assumption that the signal is generated by eudesmanolides (See Supplementary Materials).

#### 3.3.4. Semipreparative HPLC Separation

The active fractions were dissolved in 90% MeOH and injected into the Vertex Plus column (Eurospher II 100-5 C18, 250 × 8 mm) (Knauer, Berlin, Germany). The chromatographic separations were carried out in an isocratic mode (solvent flow rate 2 mL min^−1^) using MeCN: H_2_O (1:1, *v*/*v*) as the eluent. Fractions corresponding to the four major signals that showed absorption at 205 nm were collected.

#### 3.3.5. ^1^H NMR Spectroscopy

Subfractions corresponding to the signals 1 and 4, obtained by semipreparative HPLC, were subjected to ^1^H NMR analysis. NMR spectra were recorded in CDCl_3_ on a Bruker AVANCE III HD 400 (Bruker Corp., Billerica, MA, USA) (resonance frequency 400.17 MHz). Chemical shifts (δ) and coupling constants for the signals visible in the NMR spectra were compared to those of igalan (Peak 1) and alloalantolactone (Peak 4) [136,137], identified earlier by Huo et al. [114]. See Supplementary Materials (Figures S1–S5). Canonical and isomeric SMILES (Simplified Molecular-Input Line-Entry System) were derived from PubChem and input to draw the chemical structures online using the PubChem sketcher tool (Version 2.4, NCBI, Rockville, MD, USA) (Figure 3).

## 4. Conclusions

The natural product compounds attributing to the anti-staphylococcal activity of a traditional hydro-ethanolic extract of the root of *I. helenium* L. (elecampane), previously observed within our laboratory, were investigated in this study. A novel clean-up strategy resulted in a subset of (bio)active fractions, the composition of which were later analysed using a HPLC-DAD method, supported by ^1^H NMR. Based on the study by Huo et al. [114], the compounds identified using HPLC were the eudesmanolides alantolactone **(1)** and isoalantolactone **(2)**, as major constituents, and the elemanolide **(3)** igalan, plus an unseparated mixture of the eremophilanolide and eudesmanolide, dugesialactone **(4)** and alloalantolactone **(5)**, respectively. Alloalantolactone **(5)** was later confirmed, following ^1^H NMR analysis (see Supplementary Materials). Furthermore, our findings suggest that the geographical origin of the plant did not appear to influence either the chemical profile or the bioactivity of the root extract.

Elecampane clearly demonstrates activity against *Staphylococcus* spp. Considering the prevalence of MRSA and occurrence of broad-spectrum/pan-resistance in Irish hospitals amongst this Genus, further investigation into the usage of the identified SLs compounds as potential candidates in the control of staphylococcal carriage and infection is warranted. Follow-on studies could include large-scale purification or synthesis of the identified compounds, followed by in vivo analysis of the compounds, individually and in combination, as well as combinatorial experimentation to explore the possibility of utilising these compounds as potentiator or adjuvant compounds, to enhance treatment with conventional antibiotic classes.

## Figures and Tables

**Table 2:** Average zone diameter ($\bar{x}$, mm) and total yield (mg) per bioactive fraction (*n* = 3).

Extract	Bioactive Fraction No.	Inhibitory Zone Diameter ($\bar{x}$ ± SD; mm)	Total Yield ($\bar{x}$ mg)
CT50	F14	12.2 ± 0.2	13.0
	F15	16.5 ± 0.3	110.0
	F16	16.3 ± 0.5	47.7
	F17	16.1 ± 0.3	56.1
	F18	13.4 ± 0.6	55.7
	F19	10.8 ± 0.5	56.3
	F20	11.0 ± 0.3	76.3
	F21	11.5 ± 0.6	82.8
	F22	13.0 ± 0.5	96.0
CM50	F16	15.0 ± 0.6	84.7
	F17	15.6 ± 1.1	49.0
	F18	15.0 ± 0.1	42.3
	F19	13.4 ± 0.3	41.5
	F20	12.3 ± 1.2	44.3
	F21	13.1 ± 0.7	62.4
	F22	15.1 ± 0.8	43.3
	F23	16.2 ± 0.1	114.7
	F24	14.0 ± 0.9	134.4
CT100	F15	12.2 ± 0.5	44.0
	F16	13.8 ± 0.1	57.1
	F17	20.0 ± 0.1	102.5
	F18	18.7 ± 0.4	165.7
	F19	20.0 ± 0.3	84.3
	F20	12.3 ± 1.0	54.1
	F21	11.3 ± 0.2	50.5
	F22	11.2 ± 0.3	57.4
	F23	13.4 ± 0.3	57.6
CM100	F16	18.7 ± 0.7	46.1
	F17	17.4 ± 0.6	101.3
	F18	17.7 ± 1.6	61.7
	F19	14.7 ± 0.7	13.9
	F20	13.1 ± 0.1	9.7
	F21	16.5 ± 0.5	12.7

**Table 3:** Composition of bioactive fractions expressed as % of the total weight (i.e., g/100 g sample).

Extract	Fraction	Peak 1 ^a^	Peak 2	Peak 3	Peak 4	Total Yield ^b^
CT50	F14	1.64	9.72	15.91	3.52	30.79
	F15	2.85	14.62	21.07	4.07	42.62
	F16	2.89	12.52	16.32	3.51	35.24
	F17	0.79	3.81	5.87	1.10	11.56
	F18	1.87	8.93	14.32	2.66	27.78
	F19	1.34	5.92	9.24	1.86	18.35
	F20	0.20	1.10	1.75	0.34	3.40
	F21	0.40	1.68	2.35	0.52	4.95
	F22	1.64	9.72	15.91	3.52	30.79
CM50	F16	1.21	11.34	20.73	1.99	35.27
	F17	2.42	20.48	21.16	2.83	46.89
	F18	1.60	11.47	15.43	1.88	30.37
	F19	1.31	9.06	10.69	1.44	22.50
	F20	0.67	5.28	5.29	0.72	11.96
	F21	0.49	3.34	4.23	0.58	8.64
	F22	1.25	9.93	10.04	1.45	22.67
	F23	0.68	5.50	4.79	0.73	11.70
	F24	0.36	2.32	2.22	0.35	5.25

^a^ 1: Igalan ([114]; not unequivocally confirmed by ^1^H NMR (proton nuclear magnetic resonance) due to substantial amounts of lipids in the subfraction); 2: Isoalantolactone (confirmed with standard); 3: Alantolactone (confirmed with standard); 4: unseparated mixture of dugesialactone and alloalantolactone ([114] alloalantolactone presence confirmed by ^1^H NMR). ^b^ Except for the eudesmanolides, fatty acids and, as it was shown by ^1^H NMR analysis, complex mixture of lipids without UV/Vis absorption may constitute the sample.

**Table 4:** Composition of the cultivated (CT) and commercial (CM) extracts and yields.

Extract	Traditional Extract Composition	Total Yield * (g)
CT50	Cultivated root powder in 50% ethanol (*v*/*v*).	36.3
CT100	Cultivated root powder in absolute ethanol.	47.4
CM50	Commercially acquired root powder in 50% ethanol (*v*/*v*).	40.0
CM100	Commercially acquired root powder in absolute ethanol.	38.4

* Yield measured by weighing the dried residue after evaporation.

## Data Availability

The data presented in this study are contained within this article.

## Supplementary Materials

Figure S1: ^1^H NMR spectrum of isoalantolactone (in CDCl_3_). Figure S2: ^1^H NMR spectrum of isoalantolactone/alantolactone mixture isolated from roots of *Inula helenium* L. (in CDCl_3_). Figure S3: ^1^H NMR spectrum of the fraction corresponding to the peak **4** (CDCl_3_, part A expanded). Signals derived from alloalantolactone (1-deoxyivangustin) are marked with asterisks. Figure S4: ^1^H NMR spectrum of the fraction corresponding to the peak **4** (CDCl_3_, part B expanded). Signals derived from alloalantolactone (1-deoxyivangustin) are marked with asterisks. Figure S5: ^1^H NMR spectrum of the fraction corresponding to the peak **1** (CDCl_3_, part A expanded). Figure S6: ^1^H NMR spectrum of the fraction corresponding to the peak **1** (CDCl_3_, part B expanded).

## Sample Availability

Samples of the compounds are available from the authors.
