# Supplementary material for: From Monographs to Chromatograms: The Antimicrobial Potential of Inula helenium L. (Elecampane) Naturalised in Ireland
Source: Molecules. 2022 Feb 18;27(4):1406. doi: 10.3390/molecules27041406 (PMC8874828; doi:10.3390/molecules27041406)
Supplement: Supplementary file 1 [file molecules-27-01406-s001.zip › molecules-1555372-supplementary.pdf]

molecules

MDPI

Supplementary Material

From Monographs to Chromatograms: The Antimicrobial Potential of *Inula Helenium* L. (Elecampane) Naturalised in Ireland

Ciara-Ruth Kenny [1], Anna Stojakowska [2], Ambrose Furey [1,3] and Brigid Lucey [1,*]

[1] Centre for Research in Advanced Therapeutic Engineering and BioExplore, Department of Biological Sciences, Munster Technological University, Rossa Avenue, Bishopstown, T12 P928 Cork, Ireland; ciara-ruth.kenny@mycit.ie (C.-R.K.); ambrose.furey@mtu.ie (A.F.); brigid.lucey@mtu.ie (B.L.)
[2] Maj Institute of Pharmacology, Polish Academy of Sciences, 31-343 Kraków, Poland; stoja@if-pan.krakow.pl
[3] Mass Spectrometry Group, Department of Physical Sciences, Munster Technological University, Rossa Avenue, Bishopstown, T12 P928 Cork, Ireland
* Correspondence: brigid.lucey@mtu.ie; Tel.: +353-21-4335484.

Figures:

Figure S1. ^{1}H NMR spectrum of isoalantolactone (in $CDCl_3$).

Figure S2. ^{1}H NMR spectrum of isoalantolactone/alantolactone mixture isolated from roots of *Inula helenium* L. (in $CDCl_3$).

Figure S3. ^{1}H NMR spectrum of the fraction corresponding to the peak **4** ($CDCl_3$, part A expanded). Signals derived from alloalantolactone (1-deoxyivangustin) are marked with asterisks.

Figure S4. ^{1}H NMR spectrum of the fraction corresponding to the peak **4** ($CDCl_3$, part B expanded). Signals derived from alloalantolactone (1-deoxyivangustin) are marked with asterisks.

Figure S5. ^{1}H NMR spectrum of the fraction corresponding to the peak **1** ($CDCl_3$, part A expanded).

Figure S6. ^{1}H NMR spectrum of the fraction corresponding to the peak **1** ($CDCl_3$, part B expanded).

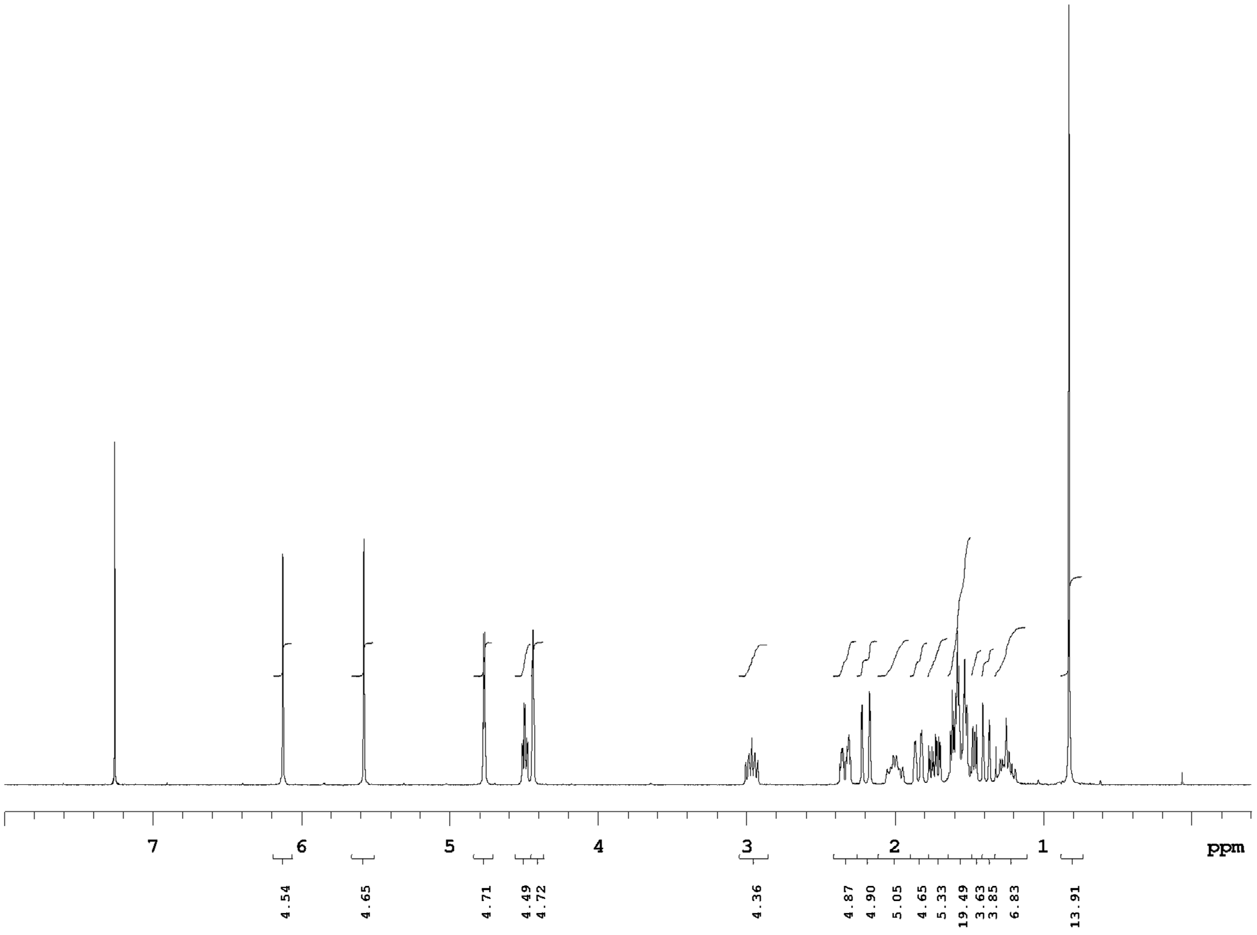

Figure S1. ^{1}H NMR spectrum of isoalantolactone.

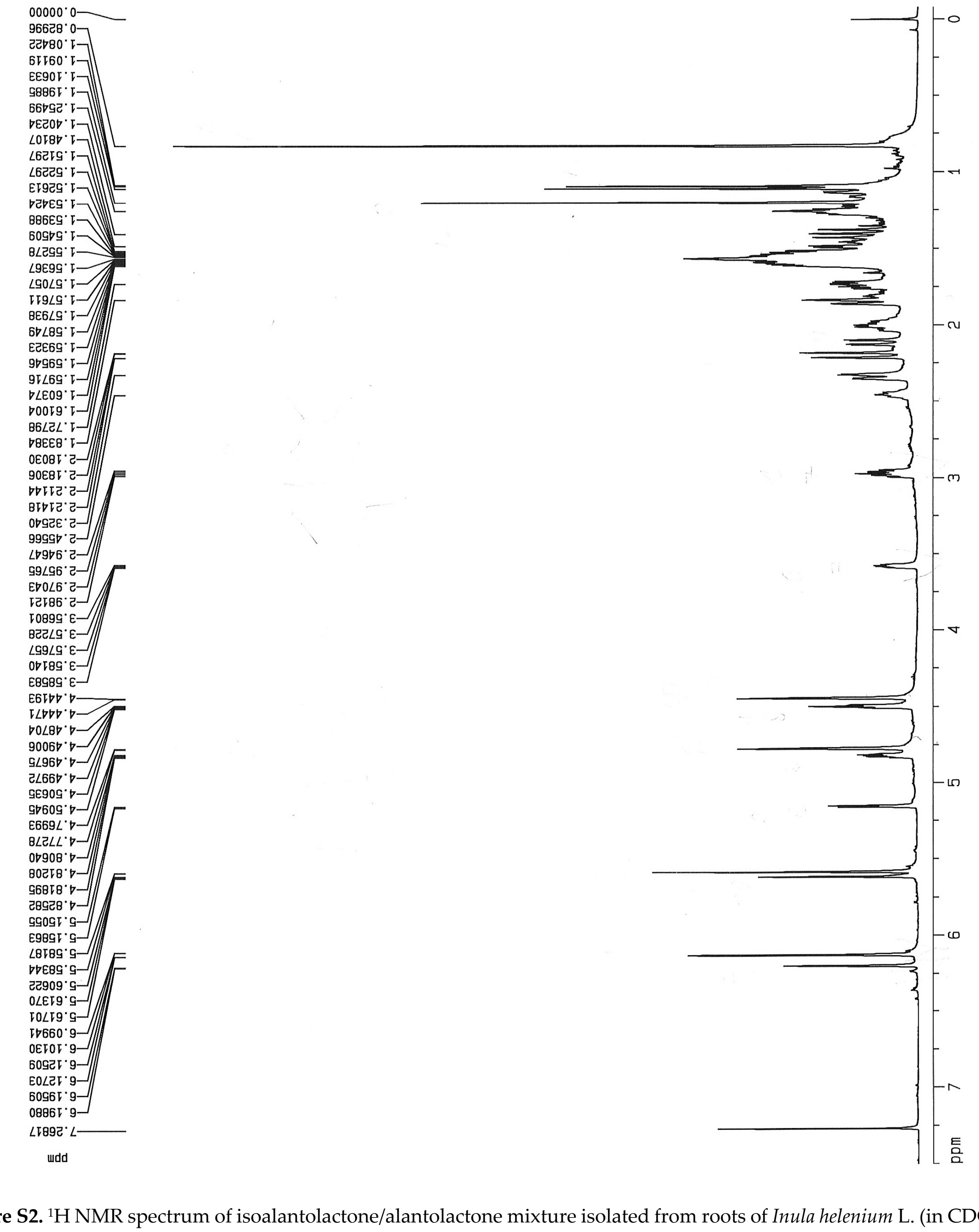

Figure S2. ^{1}H NMR spectrum of isoalantolactone/alantolactone mixture isolated from roots of *Inula helenium* L. (in $CDCl_3$).

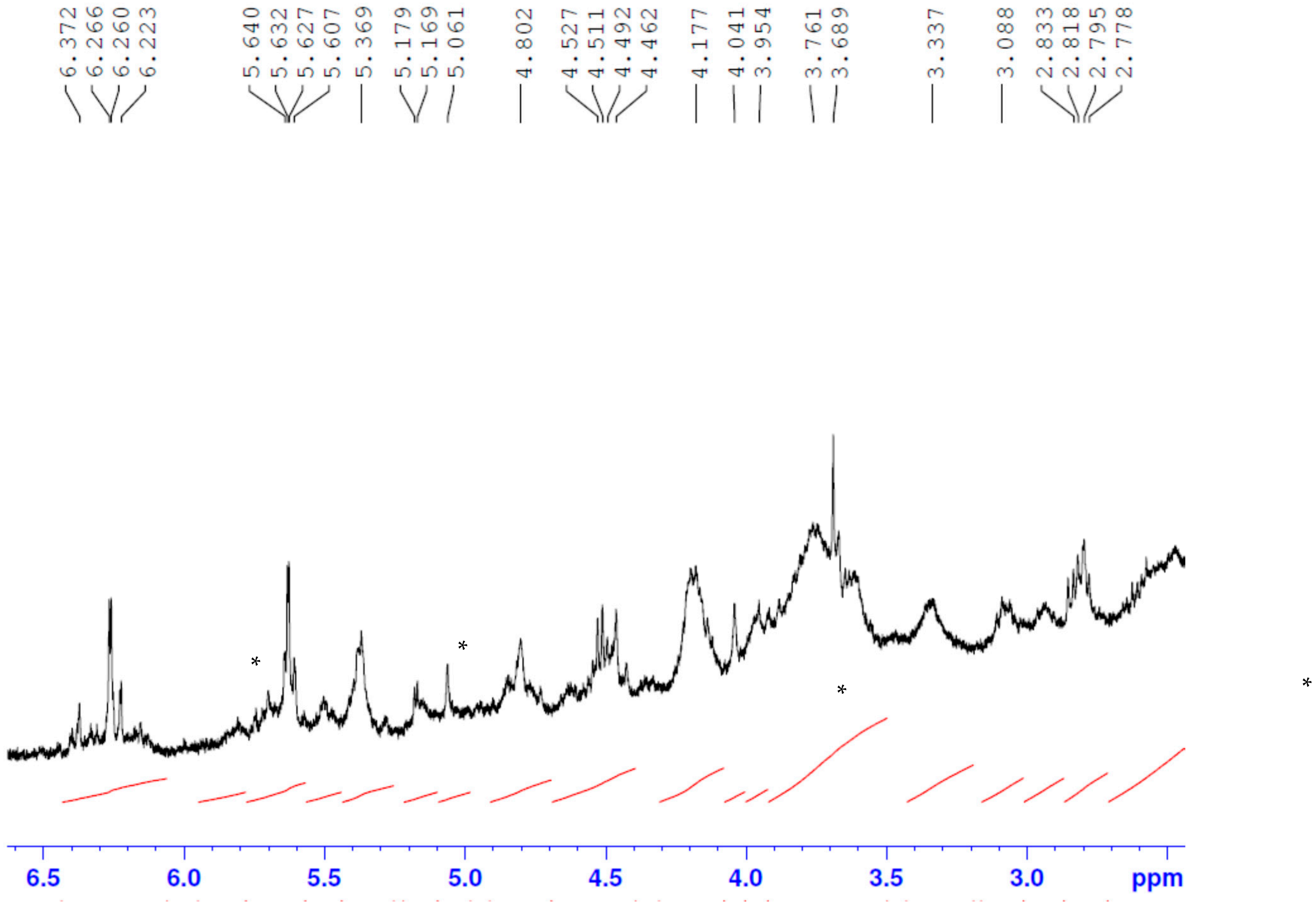

Figure S3. ^{1}H NMR spectrum of the fraction corresponding to the peak **4** ($CDCl_3$, part A expanded). Signals derived from alloalantolactone (1-deoxyivangustin) are marked with asterisks.

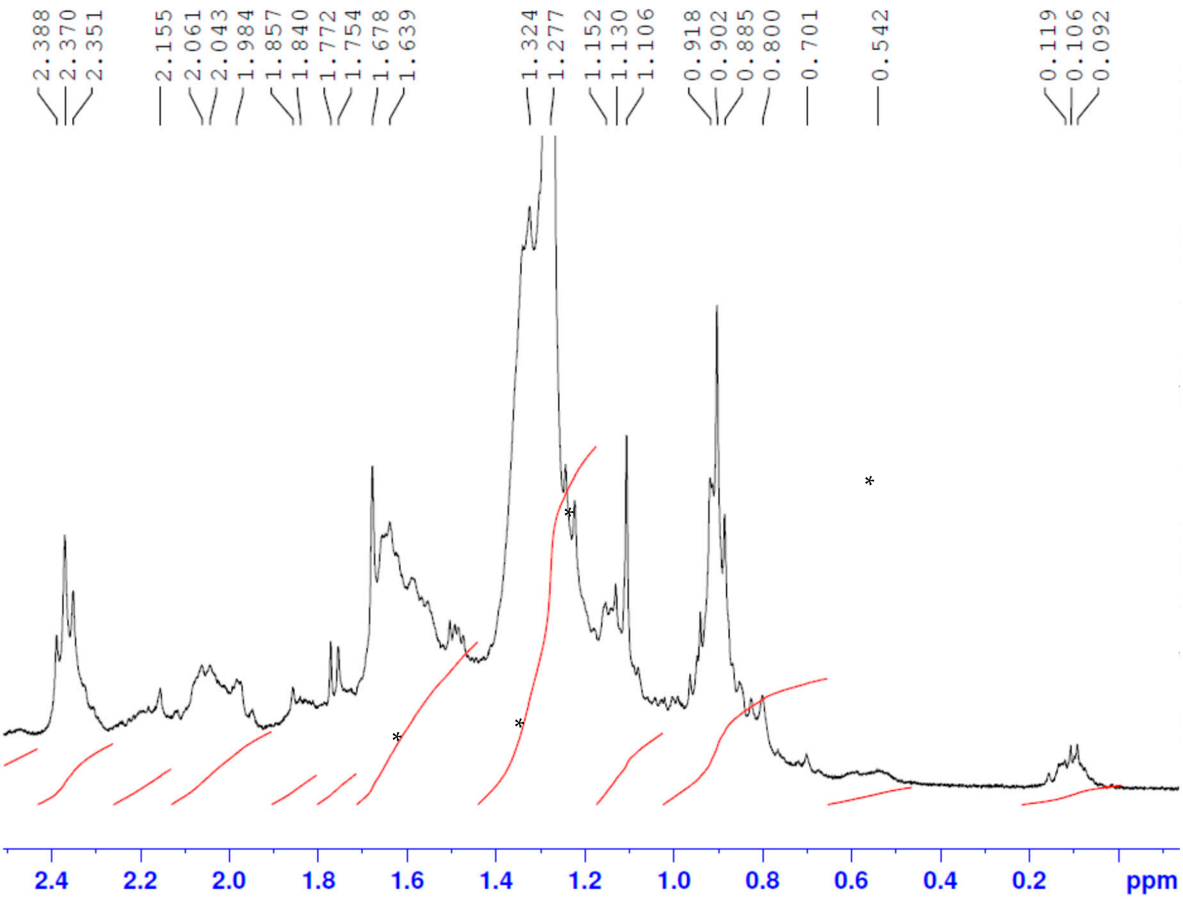

Figure S4. ^{1}H NMR spectrum of the fraction corresponding to the peak **4** ($CDCl_3$, part B expanded). Signals derived from alloalantolactone (1-deoxyivangustin) are marked with asterisks.

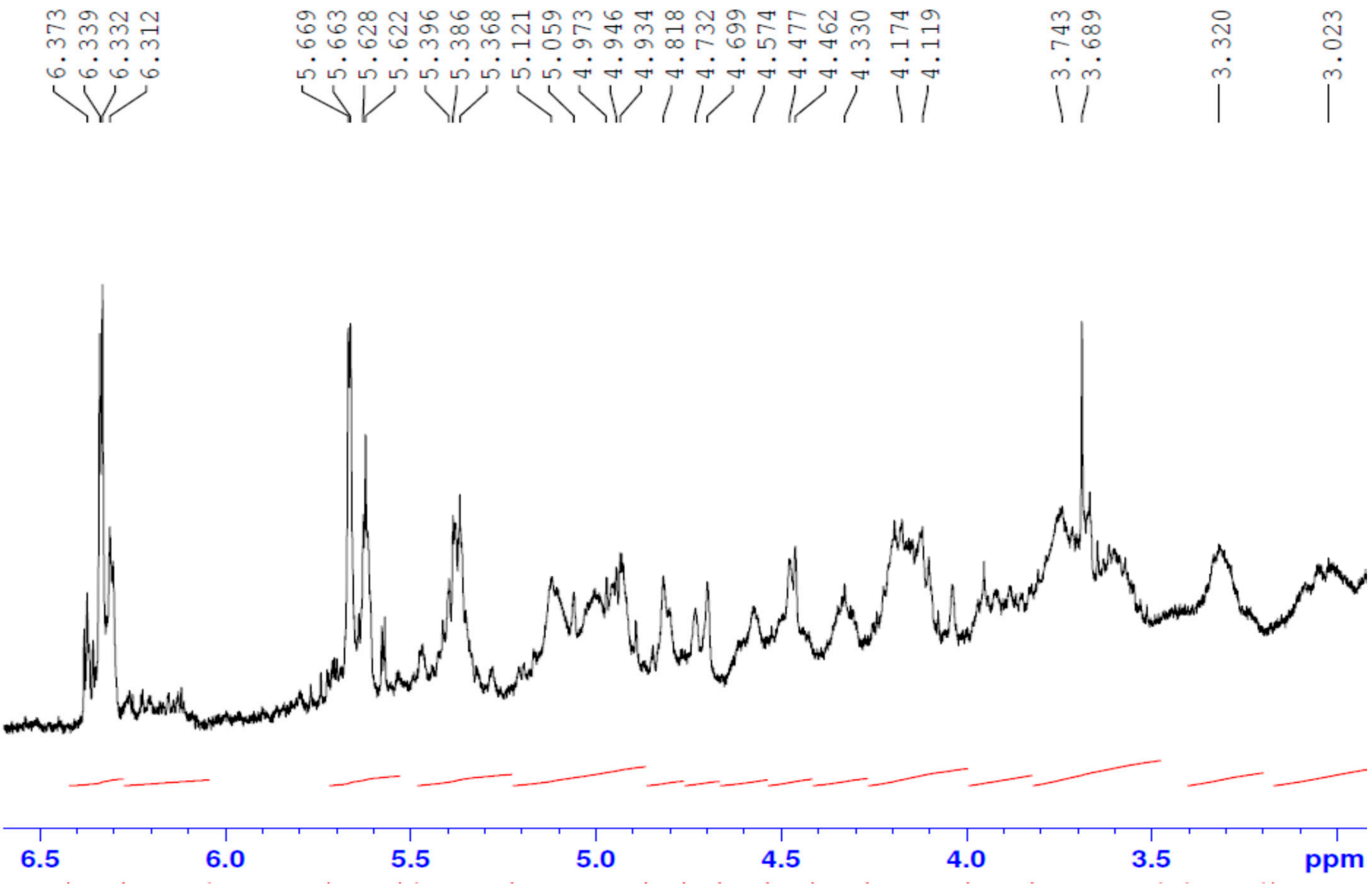

Figure S5. ^{1}H NMR spectrum of the fraction corresponding to the peak **1** ($CDCl_3$, part A expanded).

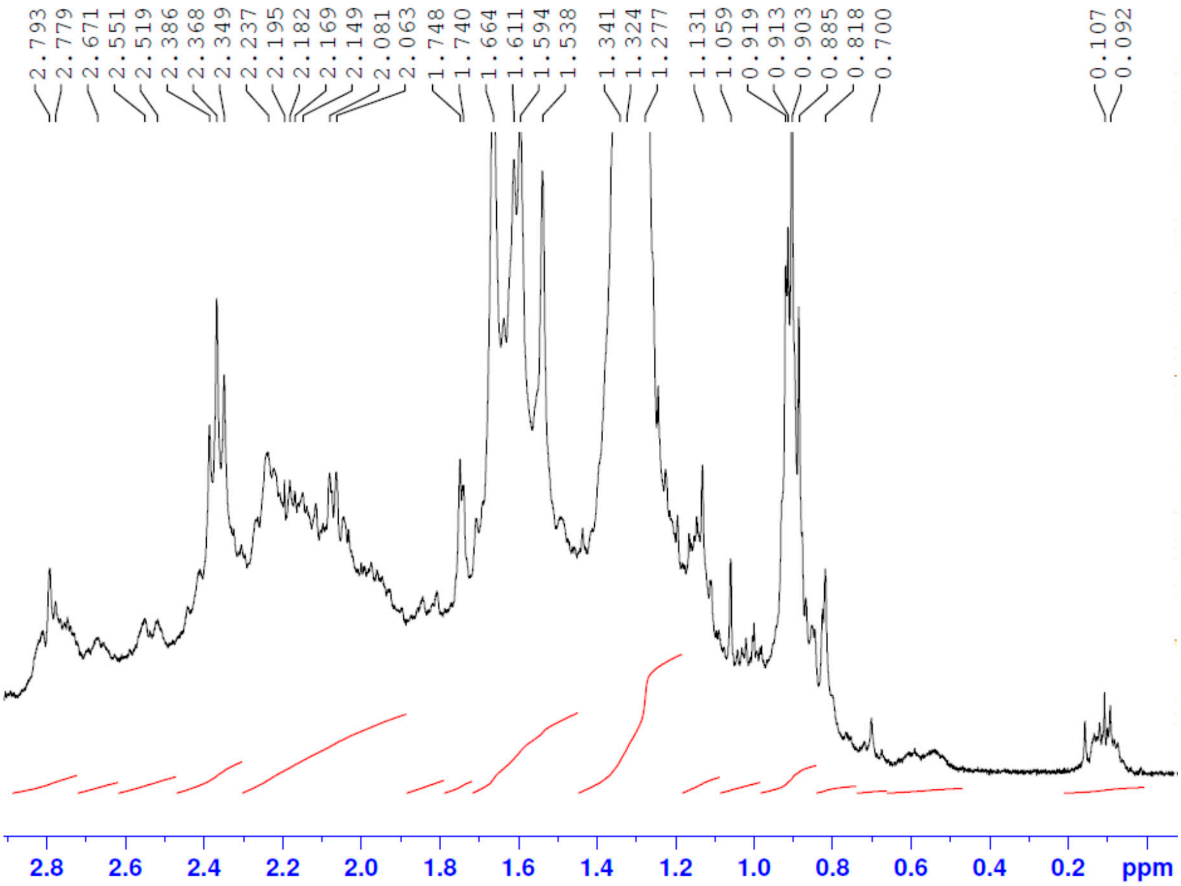

Figure S6. ^{1}H NMR spectrum of the fraction corresponding to the peak **1** ($CDCl_3$, part B expanded).
